# Molecular epidemiology and clinical features of *Klebsiella variicola* bloodstream infection compared with infection with other *Klebsiella pneumoniae* species complex strains

**DOI:** 10.1128/spectrum.03017-24

**Published:** 2025-04-25

**Authors:** Tatsuya Ohno, Sohei Harada, Hiroki Saito, Rimi Tanii, Kohji Komori, Miki Kurosawa, Haruaki Wakatake, Minoru Kanazawa, Uiri Ohki, Ayu Minoura, Mario Yamada, Satsuki Kaneko, Momoko Anzai, Yuto Tsutsui, Asuka Kuhara, Tadatomo Oyanagi, Yosuke Tanaka, Hiromu Takemura, Hiroaki Takeuchi, Hiroyuki Kunishima

**Affiliations:** 1 Department of Clinical Laboratory, St. Marianna University Yokohama Seibu Hospital 157379, Yokohama, Kanagawa, Japan; 2 Department of Medical Laboratory Sciences, Health and Sciences, International University of Health and Welfare Graduate School, Kouzunomori, Narita, Japan; 3 Department of Microbiology and Infectious Diseases, Toho University School of Medicine, Tokyo, Japan; 4 Department of Emergency and Critical Care Medicine, St. Marianna University School of Medicine 12927, Kawasaki, Kanagawa, Japan; 5 Interdepartmental Division of Critical Care Medicine, University of Toronto 7938, Toronto, Ontario, Canada; 6 Department of Clinical Laboratory Technology, St. Marianna University Hospital, Kawasaki, Kanagawa, Japan; 7 Division of Cardiovascular Medicine Saitama Medical Center, Jichi Medical University, Omiya-ku, Saitama, Japan; 8 Department of Nursing, St. Marianna University Yokohama Seibu Hospital 157379, Yokohama, Kanagawa, Japan; 9 Department of Microbiology, St. Marianna University School of Medicine 12927, Kawasaki, Kanagawa, Japan; 10 Department of Infection diseases, St. Marianna University School of Medicine 12927, Kawasaki, Kanagawa, Japan; Laboratory Corporation of America Holdings, Burlington, North Carolina, USA

**Keywords:** *Klebsiella variicola*, *Klebsiella pneumoniae*, bloodstream infections, hypervirulent, molecular epidemiology

## Abstract

*Klebsiella variicola* is an emerging pathogen within the *Klebsiella pneumoniae* species complex, and its clinical and microbiological characteristics remain poorly understood. This retrospective case-control study analyzed 252 patients with bloodstream infections caused by *K. pneumoniae* species complex, including 60 with *K. variicola* infection, to elucidate these characteristics. Our study showed no significant differences in clinical outcomes, such as 30-day mortality, between *K. variicola* and *K. pneumoniae*. However, a significant difference was found in the rate of harboring [*peg-344*, *iucA*, and *rmpA*] genes, which are associated with virulence in *K. pneumoniae*, suggesting that *K. variicola* may be generally less virulent. Notably, we identified two patients with community-acquired liver abscess caused by hypervirulent *K. variicola*, representing the first genetically analyzed case of this phenomenon in Japan and highlighting the potential virulence of this species. While there have been several reports on *K. variicola* carrying hypervirulence genes, this is the first report in Japan, to our knowledge, to genetically characterize a hypervirulent *K. variicola* isolated from a patient with disseminated liver abscesses using whole-genome sequencing. Multilocus sequence typing revealed high diversity among *K. variicola* isolates, with 49 distinct sequence types identified, 30 of which were newly registered, highlighting the genetic heterogeneity of this pathogen. No significant clinical differences were observed between *K. variicola* and other *Klebsiella* spp. The emergence of hypervirulent *K. variicola* strains with the potential to cause severe complications warrants further surveillance and research.

**IMPORTANCE:**

*Klebsiella variicola* is increasingly recognized as an emerging pathogen commonly found in the environment and human gut. However, its clinical and microbiological characteristics remain poorly understood. This study provides a comprehensive analysis of *K. variicola* bloodstream infections (BSIs), comparing clinical and genetic features with the closely related *K. pneumoniae*. We identified significant differences in the prevalence of virulence genes between the two species. Notably, we observed *K. variicola* causing disseminated liver abscesses, similar to hypervirulent *K. pneumoniae* strains. These findings have important implications for accurate species identification, informing treatment strategies, and improving patient outcomes in the face of this emerging infectious threat.

## INTRODUCTION

The *Klebsiella pneumoniae* species complex comprises seven phylogroups: *K. pneumoniae*, *K. quasipneumoniae* subsp. *quasipneumoniae*, *K. quasipneumoniae* subsp. *similipneumoniae*, *K. variicola* subsp. *variicola*, *K. variicola* subsp. *tropica*, and *K. africana*. The most commonly identified human species are *K. pneumoniae*, *K. variicola*, and *K. quasipneumoniae* (1). In 2004, *K. variicola* was identified as a new species through genetic and biochemical analyses (2). *K. variicola*, similar to *K. pneumoniae*, causes infectious diseases such as bloodstream infections (BSIs), respiratory tract infections, and urinary tract infections (3).

Several studies have demonstrated an association between clinical characteristics and species within the *K. pneumoniae* species complex (4–6). Although *K. pneumoniae* is the most prevalent cause of BSI within this complex, *K. variicola* has been linked to higher mortality rates (4). However, the relationship between bacterial species and clinical outcomes, including mortality, remains controversial. Additionally, we previously reported a fatal case of coronavirus disease 2019 complicated by rapidly progressive sepsis caused by infection with *K. variicola* (7). Furthermore, patients with *K. variicola* BSI exhibiting a hypermucoviscous phenotype associated with hypervirulence in *K. pneumoniae* have been documented (8–11). However, the virulence genes harbored by *K. variicola*, their role in the development of phenotypic manifestations, and the molecular epidemiology of this organism have not been well described in the literature. This study aimed to address this knowledge gap by comprehensively analyzing the clinical and molecular epidemiology of *K. variicola* infection in Japan.

## MATERIALS AND METHODS

### Participants and bacterial isolates

This retrospective cohort study included all patients admitted to St. Marianna University Yokohama Seibu Hospital and St. Marianna University Hospital (Kanagawa, Japan) between 2018 and 2021 who were diagnosed with BSI caused by the *K. pneumoniae* species complex. For patients with multiple positive blood cultures for the same organism within 90 days, the data from the first positive blood culture were used. A clinical microbiology laboratory information system was used to identify the isolates and patients. Species identification was performed using matrix-assisted laser desorption/ionization (MALDI) Biotyper with Reference Library version 9 (Bruker Daltonics, Bremen, Germany).

### Collection of clinical data

Clinical data were collected from the medical records, including age, sex, vital signs, laboratory findings, comorbidities based on the Charlson Comorbidity Index (12), hemodialysis status, source of BSI, time to initiation of appropriate antimicrobial therapy, and 30-day mortality. The origin of the infection was classified as either community acquired (including those contracted in nursing homes or long-term care facilities) or hospital acquired. Hospital-acquired infections were defined as infections identified by positive blood cultures obtained more than 48 h after admission. Appropriate antimicrobial therapy was defined as the use of antimicrobials to which the isolates were susceptible, as determined by *in vitro* susceptibility testing. An infection was classified as polymicrobial if at least one or more different species were detected in the blood along with the *K. pneumoniae* species complex. Skin-contaminating bacteria such as *Corynebacterium* spp., *Bacillus* spp., *Cutibacterium* spp., and coagulase-negative *Staphylococcus* spp. were treated as causative pathogens if at least two separate sets of blood cultures yielded positive results. By contrast, the detection of other species in a single blood culture was deemed sufficient (13). Continuous BSI was defined as the detection of the same organism in a blood culture taken on or after the day following the initial detection of that organism in a patient receiving a susceptible antimicrobial. Disseminated infection was defined as the involvement of multiple infected organs. The quick Pitt bacteremia (qPITT) and Sequential Organ Failure Assessment (SOFA) scores were used to evaluate disease severity. The q-PITT score, based on temperature, blood pressure, respiratory rate, cardiac arrest, and mental status, allows for rapid bedside assessment. The SOFA score, using laboratory data to assess six organ systems, provides a more detailed evaluation of organ dysfunction.

### Microbiological and molecular analyses

#### Identification of *K. pneumoniae* species complex by multiplex polymerase chain reaction

The *K. pneumoniae* species complex was identified using a multiplex polymerase chain reaction (PCR) assay as previously reported (14). DNA templates were prepared using Cica Geneus DNA extraction reagent (Kanto Chemical Co., Inc., Tokyo, Japan) and primers used in previous studies. The PCR mixtures contained 9.25 µL of TaKaRa *Taq* hot-start (HS) version (*Takara Taq* HS: 0.25 µL, 10 × PCR buffer: 5 µL, deoxynucleotide triphosphate (dNTP) mixture: 4 µL; Takara Bio Inc., Gunma, Japan), 5 µL of template DNA, 2 µL each of forward and reverse primers, and 31.75 µL of distilled water, with a total reaction volume of 50 µL. PCR was performed using the Gene Atlas E02 Economy thermal cycler (ASTEC Co., Ltd., Osaka, Japan) under the following conditions: an initial denaturation for 5 min at 95°C, followed by 40 amplification cycles (30 s at 94°C, 30 s at 55°C, and 60 s at 72°C), with a final extension step of 10 min at 72°C. The PCR products were subjected to electrophoresis on a 2% agarose gel (Kanto Chemical Co., Inc., Tokyo, Japan) in 1 × Tris-acetate-ethylenediaminetetraacetic acid (TAE) buffer at 100 V for 45 min. The amplicon size was estimated using a 100 bp molecular weight marker and stained with ethidium bromide (Kanto Chemical Co., Inc., Tokyo, Japan). The primers used are listed in Table S1.

#### Adonitol hydrolysis test

Peptone (1.5 g), bromocresol purple (0.08%, 2.5 mL), adonitol (10%, 5.0 mL), and distilled water were combined to a total volume of 100 mL. The solution was then dispensed into sterile tubes in 2 mL portions and subjected to high-pressure steam sterilization. Following inoculation with the test organisms, the tubes were incubated at 37°C, and a yellow color change was considered a positive result.

#### String test

The hypermucoviscous phenotype of the isolates was determined using the string test (15, 16). Mucopurulent strings were extended from each colony and cultured overnight on 5% sheep blood agar using a 1 µL loop. Mucopurulent strings at least 5 mm in length were used as positive criteria.

#### Mucoviscosity assay test

For *K. variicola* isolates with a positive string test, the degree of sedimentation was assessed as described in a previous study (17). Overnight cultures were pelleted by centrifugation at 9,400 *g* and resuspended in phosphate-buffered saline to an OD_600_ of approximately 1. The suspension was centrifuged at 1,000 *g* for 5 min, and the OD_600_ of the supernatant was measured.

#### Antimicrobial susceptibility test

Antimicrobial susceptibility testing was performed using the MicroScan WalkAway system (Beckman Coulter, Brea, CA, USA). Potential extended-spectrum beta-lactamase (ESBL) and/or AmpC producers were confirmed using AmpC/ESBL differential discs (Kanto Chemical Co. Inc., Tokyo, Japan). The minimum inhibitory concentrations were determined according to the guidelines of the Clinical and Laboratory Standards Institute (M100-S33) (18).

### Detection of virulence factor genes

The hypervirulence factor-encoding genes, *peg-344* (metabolite transporter), *iucA* (aerobactin siderophore biosynthesis), and *rmpA* (regulator of mucoid phenotype A), were detected by PCR using the previously reported primers (19). DNA templates were prepared using the Cica Geneus DNA extraction reagent (Kanto Chemical Co., Inc., Tokyo, Japan). The PCR mixtures contained 9.25 µL of TaKaRa Taq HS version (Takara Taq HS: 0.25 µL, 10 × PCR buffer: 5 µL, and dNTP mixture: 4 µL; Takara Bio Inc., Gunma, Japan), 5 µL of template DNA, 2 µL each of forward and reverse primers, and 31.75 µL of distilled water, with a total reaction volume of 50 µL. PCR was performed using a Gene Atlas E02 Economy thermal cycler (ASTEC Co., Ltd., Osaka, Japan) under the following conditions: an initial denaturation at 95°C for 2 min, followed by 25 amplification cycles of 95°C for 30 s, primer-specific annealing temperature for 30 s, and 72°C for 40 s, with a final extension at 72°C for 10 min. The PCR products were subjected to electrophoresis on a 2% agarose gel (Kanto Chemical Co., Inc., Tokyo, Japan) in 1 × TAE buffer at 100 V for 45 min. The amplicon size was estimated using a 100 bp molecular weight marker and stained with ethidium bromide (Kanto Chemical Co., Inc., Tokyo, Japan). Presumptive hypervirulent (p-hv) strains were defined as those harboring one or more of the *peg-344*, *iucA*, or *rmpA* genes (19, 20). The primers used are listed in Table S1.

### Multilocus sequence typing and phylogenetic analysis of *K. variicola* isolates

The sequence type (ST) was determined using the *K. variicola* MLST scheme (https://mlstkv.insp.mx/) (21). This scheme analyzed seven housekeeping genes (*leuS*, *pgi*, *pgk*, *phoE*, *pyrG*, *rpoB*, and *fusA*) to assign a unique ST to each *K. variicola* isolate.

A maximum likelihood phylogenetic tree was constructed using the concatenated sequences of seven housekeeping genes in MEGA 11 (https://www.megasoftware.net/). The reliability of the tree was assessed using bootstrap analysis (500 replicates), and a general time-reversible model was employed to calculate genetic distances.

### Whole-genome sequencing of *K. variicola* strains with cardinal virulence genes

The genomic sequences of the p-hv *K. variicola* strains, identified according to the definition mentioned above, were analyzed using the following method. DNA was extracted from bacterial cells using magLEAD 6gC (Precision System Science Co., Ltd., Chiba, Japan) following the MagDEA Dx SV PS protocol. DNA libraries were prepared using an Illumina DNA Prep kit (Illumina, Inc., San Diego, CA, USA) and sequenced on a MiSeq (Illumina) with 300 bp paired-end reads. The raw reads generated by MiSeq were adapter trimmed and quality filtered using the Trimmomatic tool (version 0.39), with a quality cutoff score of Q30, and assembled using SPAdes (version 3.15.4). The genomic features of the strains were characterized using Kaptive and Kleborate available on the Pathogenwatch website (https://pathogen.watch/). The comprehensive identification of the virulence genes from the *K. pneumoniae* species complex was performed using the *Klebsiella* locus/sequence definition database available on the BIGSdb website (https://bigsdb.pasteur.fr/klebsiella/). The programs were run using default parameters.

### Statistical analysis

Categorical variables were expressed as proportions (%), and comparisons between the two groups were carried out using Fisher’s exact test. Continuous variables were expressed as medians and interquartile ranges (IQRs), and comparisons between the two groups were performed using the Wilcoxon rank-sum test. Multivariate logistic regression analysis was used to assess the association between the independent risk factors and mortality. Factors that were significantly associated with mortality in the univariate analysis were included in the initial multivariate analysis. To further refine the model, additional clinically and microbiologically important factors were also included in the analysis: disseminated infection, continuous bacteremia, appropriate antibiotic therapy, and age. These factors were included in the analysis and adjusted for because they were thought to be potential confounders of the relationship between *K. variicola* infection and mortality. For all tests, a two-tailed P-value of <0.05 was considered significant. Statistical analyses were performed using the JMP 15.2 software (SAS Institute, Inc., Cary, NC, USA).

## RESULTS

### Clinical characteristics

The clinical characteristics of patients with BSI caused by *K. variicola*, *K. pneumoniae*, or *K. quasipneumoniae* are summarized in Table 1. The median age of the 252 patients was 77 years (IQR: 68–83 years), with 94 (37.3%) patients aged >80 years. Of the 252 patients, 66.3% (*n* = 167) were men. The most common underlying diseases were solid tumors (*n* = 86, 34.1%) and diabetes mellitus (*n* = 65, 25.8%). The primary sources of infection were biliary tract infection or cholecystitis in 88 patients (34.9%) and urinary tract infection in 52 patients (20.6%). Liver abscesses were observed in 14 patients (5.6%). Among the 252 patients, 214 (84.9%) received appropriate antimicrobial therapy within 24 h. Hospital-acquired infections were identified in 33.3% (84) of the patients. Continuous bacteremia and disseminated infection were present in 10 (4%) and 7 (2.8%) patients, respectively. The median qPITT and SOFA scores were 0 (IQR: 0–1) and 3 (IQR: 2–5.5), respectively. The 30-day mortality rate was 19.1% (*n* = 48).

**TABLE 1:** Clinical characteristics of patients with bloodstream infections caused by *K. variicola, K. pneumoniae, and K. quasipneumoniae^d^*

Characteristic	ALL (*n* = 252) No (%)		*K. variicola*^*a*^ (*n* = 60) No (%)		*K. pneumoniae*^*a*^ (*n* = 178) No (%)		*K. quasipneumoniae*^*a*^ (*n* = 14) No (%)		*K. variicola* vs Other *Klebsiella* spp. *P*-value^*b*^
Age (years) (median〔IQR^*c*^〕）	77	(68–83)	77.5	(67.25–85)	77	(67.75–83)	77.5	(72.5–80.75)	0.628
Age >80 years	94	(37.3)	24	(40.0)	66	(37.1)	4	(28.6)	0.648
Male sex	167	(66.3)	40	(66.7)	118	(66.3)	9	(64.3)	1
Body temperature (median〔IQR^*c*^〕）	38.2	(37–38.9)	38.3	(37–39)	38.2	(37.1–39)	37	(36.5–38.2)	0.805
WBC (×10^3^/µL) (median〔IQR^*c*^〕）	9.5	(5.9–13.8)	10.2	(7.1–14.6)	9.3	(5.28–13.8)	9.8	(4.6–12.7)	0.131
Plt (×10^4^/µL) (median〔IQR^*c*^〕）	16.6	(10.8–22.9)	17.6	(11.6–22)	16.5	(10.3–23.1)	11.5	(6.5–18.6)	0.678
CRP (mg/dL) (median〔IQR^*c*^〕）	7.5	(2.3–17.4)	9	(2.7–22.4)	6.92	(2.2–15.9)	7.1	(1.6–19.9)	0.098
Comorbidities									
Charlson index (median 〔IQR^*c*^〕)	2	(1–3)	2	(1, 2)	2	(1–3)	2.5	(0.75–4)	
Diabetes mellitu	65	(25.8)	11	(18.3)	54	(30.3)	0	(0)	0.176
Solid tumors	86	(34.1)	24	(40)	58	(32.6)	4	(28.6)	0.279
Liver cirrhosis	15	(6)	3	(5)	11	(6.2)	1	(7.1)	1
Collagen disease	5	(2)	0	(0)	5	(2.8)	0	(0)	0.595
Kidney disease	20	(7.9)	1	(1.7)	15	(8.4)	4	(28.6)	0.052
Pulmonary disease	7	(2.8)	1	(1.7)	6	(3.4)	0	(0)	1
Hematological malignancy	18	(7.1)	2	(3.3)	14	(7.9)	2	(14.3)	0.257
Immunosuppressive drug	36	(14.3)	5	(8.3)	30	(16.9)	1	(7.1)	0.145
Neutropenia	8	(3.2)	0	(0)	7	(3.9)	1	(7.1)	0.204
Hemodialysis	9	(3.6)	3	(5)	6	(3.4)	0	(0)	0.448
Source of infection									
Biliary tract/cholecystitis	88	(34.9)	27	(45)	54	(30.3)	7	(50)	0.065
Liver abscess	14	(5.6)	4	(6.7)	10	(5.6)	0	(0)	0.747
Intra-abdominal	20	(7.9)	8	(13.3)	12	(6.7)	0	(0)	0.099
Intravenous catheter related	11	(4.3)	1	(1.7)	9	(5.1)	1	(7.1)	0.468
Respiratory tract	25	(9.9)	4	(6.7)	21	(11.8)	0	(0)	0.460
Skin and soft tissue	3	(1.2)	0	(0)	3	(1.7)	0	(0)	1
Urinary tract	52	(20.6)	10	(16.7)	38	(21.4)	4	(28.6)	0.467
Others	4	(1.6)	0	(0)	4	(2.2)	0	(0)	0.575
Unknown	35	(13.9)	6	(10)	27	(15.2)	2	(14.3)	0.396
Appropriate antibiotic therapy within 24 h	214	(84.9)	50	(83.3)	151	(84.8)	13	(92.9)	0.683
Hospital-acquired infection	84	(33.3)	24	(40)	59	(33.2)	1	(7.1)	0.214
Continuous bacteremia	10	(4)	4	(6.7)	6	(3.4)	0	(0)	0.255
Disseminated infection	7	(2.8)	1	(1.7)	6	(3.4)	0	(0)	1
q-PITT score (median〔IQR^*c*^〕）	0	(0–1)	0	(0–1)	0	(0–1)	1	(0–1)	**0.022**
SOFA score (median〔IQR^*c*^〕）	3	(2–5.5)	3	(2–5.75)	3	(2–5)	4	(2–6.25)	0.621
30-day mortality	48	(19.1)	12	(20)	35	(19.7)	1	(7.1)	0.851

^*a*^ Identification of the *K. pneumoniae* species complex was performed using multiplex polymerase chain reaction (14).

^*b*^ Results of Fisher’s exact test.

^*c*^ IQR, interquartile range.

^*d*^ Statistically significant differences are indicated in bold.

Although no significant differences were found in clinical characteristics between *K. variicola* and other *Klebsiella* spp., the qPITT score was significantly lower for *K. variicola*.

### Bacterial characteristics

The microbiological findings are summarized in Table 2. Multiplex PCR analysis identified 178 (70.6%) isolates as *K. pneumoniae*, 60 (23.8%) as *K. variicola*, and 14 (5.6%) as *K. quasipneumoniae*. One isolate was identified as *K. pneumoniae* by MALDI time-of-flight mass spectrometry (MALDI-TOF MS), tested negative for all multiplex PCR target genes, and classified under the *K. pneumoniae* group. The adonitol hydrolysis test yielded a positive result in 171 (96.1%) *K*. *pneumoniae* strains, 5 (8.3%) *K*. *variicola* strains, and 12 (85.7%) *K*. *quasipneumoniae* strains.

**TABLE 2:** The bacterial characteristics of *K. variicola*, *K. pneumoniae,* and *K. quasipneumoniae^c^*

Bacterial characteristic	ALL (*n* = 252) No (%)		*K. variicola^a^* (*n* = 60) No (%)		*K. pneumoniae^a^* (*n* = 178) No (%)		*K. quasipneumoniae^a^* (*n* = 14) No (%)		*K. variicola* vs Other *Klebsiella* spp. *P*-value^*b*^
MALDI-TOF MS									
*K. variicola*	60	(23.8)	58	(96.7)	1	(0.6)	1	(7.1)	
*K. pneumoniae*	192	(76.2)	2	(3.3)	177	(99.4)	13	(92.8)	
Adonitol hydrolysis	188	(74.6)	5	(8.3)	171	(96.1)	12	(85.7)	**<0.001**
Presumptive hypervirulent strains	40	(15.9)	2	(3.3)	38	(21.3)	0	(0)	**<0.001**
*iucA-peg344-rmpA*	23	(9.1)	1	(1.7)	22	(12.4)	0	(0)	
*iucA-peg344*	0	(0)	0	(0)	0	(0)	0	(0)	
*iucA-rmpA*	1	(0.4)	0	(0)	1	(0.6)	0	(0)	
*peg344-rmpA*	4	(1.6)	0	(0)	4	(2.2)	0	(0)	
*iucA* only	8	(3.2)	0	(0)	8	(4.5)	0	(0)	
*peg344* only	0	(0)	0	(0)	0	(0)	0	(0)	
*rmpA* only	4	(1.6)	1	(1.7)	3	(1.7)	0	(0)	
Hypermucoviscous phenotype (string test >5 mm)	48	(19)	10	(16.7)	36	(20.2)	2	(14.3)	0.708
*rmpA* gene-positive	13	(5.2)	2	(3.3)	11	(6.2)	0	(0)	
*rmpA* gene-negative	35	(13.9)	8	(13.3)	25	(14.0)	2	(14.3)	
Polymicrobial	62	(24.6)	15	(25)	44	(24.7)	3	(21.4)	1
ESBL-producing (include AmpC)	10	(4.0)	0	(0)	10	(5.6)	0	(0)	0.123

^*a*^ Identification of the *K. pneumoniae* species complex was performed using multiplex polymerase chain reaction (14).

^*b*^ Results of Fisher’s exact test.

^*c*^ Statistically significant differences are indicated in bold.

Forty (15.9%) strains tested positive for the virulence genes *peg-344*, *iucA* or *rmpA*, and were classified as p-hv strains in this study. These virulence genes were detected in 38/178 (21.3%) *K*. *pneumoniae* strains, 2/60 (3.3%) *K*. *variicola* strains, and 0/14 (0%) *K*. *quasipneumoniae* strains, indicating a significantly higher prevalence in *K. pneumoniae*.

The hypermucoviscosity phenotype, determined by a positive string test, was observed in 36 (20.2%) *K*. *pneumoniae* strains, 10 (16.7%) *K*. *variicola* strains, and 2 (14.3%) *K*. *quasipneumoniae* strains, with no significant difference.

Infections with p-hv strains were significantly associated with higher C-reactive protein (odds ratio [OR] = 1.03, 95% confidence interval [CI]: 1.00–1.07, *P* = 0.009), diabetes mellitus (OR = 2.22, 95% CI: 1.08–4.48, *P* = 0.031), liver abscesses (OR = 17.33, 95% CI: 5.43–66.46, *P* ≤ 0.001), respiratory tract infections (OR = 4.38, 95% CI: 1.76–10.57, *P* = 0.002), disseminated infections (OR = 15, 95% CI: 3.10–107.59, *P* = 0.001), and higher SOFA scores (OR = 17.33, 95% CI: 5.43–66.46, *P* ≤ 0.001) (Table 3). Among the 10 *K. variicola* strains with a hypermucoviscous phenotype, two strains tested positive for virulence genes. The two cases were associated with BSI that originated from community-acquired liver abscesses (Table S2).

**TABLE 3:** Comparison of clinical characteristics of presumptive hypervirulent *K. pneumoniae* species complex infections and those caused by other isolates^*a*^^*,^d^*^

Clinical characteristic	Presumptive hypervirulent strains (*n* = 40) No (%)		Non-presumptive hypervirulent strains (*n* = 212) No (%)		*P*-value^*b*^	Odds ratio (95% confidence interval)	
Age (years)(median〔IQR^*c*^〕）	76	(72.3–82.8)	77	(67.3–83)	0.688	1.01	(0.99–1.04)
Male sex	31	(77.5)	136	(64.2)	0.144	1.92	(0.90–4.49)
CRP (mg/dL) (median〔IQR^*c*^〕）	15.7	(3.6–20.5)	6.7	(2–15.9)	**0.009**	**1.03**	**(1.00–1.07)**
Comorbidities							
Diabetes mellitus	16	(40)	49	(23.1)	**0.031**	**2.22**	**(1.08–4.48)**
Source of infection							
Liver abscess	10	(25)	4	(1.9)	**<0.001**	**17.33**	**(5.43–66.46)**
Respiratory tract	10	(25)	15	(7.1)	**0.002**	**4.38**	**(1.76–10.57)**
Disseminated infection	5	(12.5)	2	(0.9)	**0.001**	**15**	**(3.10–107.59)**
Hospital-acquired infection	10	(25)	74	(34.9)	0.274	0.62	(0.28–1.30)
q-PITT score (median〔IQR^*c*^〕）	0	(0–2)	0	(0–1)	0.096	**1.48**	**(1.10–1.98)**
SOFA score (median〔IQR^*c*^〕）	4	(2–9)	3	(2–5)	**0.008**	**1.14**	**(1.04–1.25)**
String test	15	(37.5)	33	(15.6)	**0.003**	**3.25**	**(1.53–6.79)**
30-day mortality	12	(30)	36	(17)	0.077	2.1	(0.95–4.44)

^*a*^ A presumptive hypervirulent strain was defined as one harboring one or more of the *peg-344*, *iucA*, or *rmpA* genes (19).

^*b*^ Results of Fisher's exact test.

^*c*^ IQR, interquartile range.

^*d*^ Statistically significant differences are indicated in bold.

### Thirty-day mortality risk factor analysis

A comparison of the clinical characteristics between the fatal and survivor groups is shown in Table 4. The risk factors for 30-day mortality in the univariate analysis were neutropenia (OR = 7.79, 95% CI: 1.79–33.84, *P* = 0.007), intra-abdominal infection (OR = 4.05, 95% CI: 1.57–10.43, *P* = 0.005), respiratory tract infection (OR = 13.44, 95% CI: 5.34–33.77, *P* < 0.001), hospital-acquired infections (OR = 2.16, 95% CI: 1.14–4.09, *P* = 0.026), qPITT score (OR = 2.20, 95% CI: 1.62–3.00, *P* < 0.001), and SOFA score (OR = 1.27, 95% CI: 1.16–1.41, *P* < 0.001). No significant differences were observed in bacteriological characteristics such as species or phenotype. Multivariate analysis identified neutropenia (OR = 8.88, 95% CI: 1.38–57.2, *P* = 0.021), intra-abdominal infection (OR = 6.25, 95% CI: 2.02–19.3, *P* = 0.001), respiratory tract infection (OR = 10.37, 95% CI: 3.53–30.43, *P* < 0.001), and qPITT score (OR = 1.92, 95% CI: 1.33–2.76, *P* < 0.001) as significant predictors of 30-day mortality. Meanwhile, hospital-acquired infection was not a significant predictor in the multivariate model (OR = 1.59, 95% CI: 0.73–3.48, *P* = 0.245). No significant difference was observed in p-hv strains (OR = 1.40, 95% CI: 0.49–4.01, *P* = 0.529). The SOFA score was excluded from the multivariate analysis due to its collinearity with the qPITT score.

**TABLE 4:** Comparison between fatal group and the survivor group^*e*^

Characteristic	Fatal (*n* = 48) No (%)		Survivors (*n* = 204) No (%)		*P-value^a^*	Odds ratio (95% confidence interval)			
						Univariate analysis		Multivariate analysis^*b*^	
Age (years) (median〔IQR^*c*^〕）	79	(63.3–84.8)	77	(68–83)	0.318	1.01	(0.99–1.03)	1.01	(0.98–1.04)
Age >80 years	23	(47.9)	71	(34.8)	0.099	1.72	(0.91–3.25)		
Male sex	28	(58.3)	139	(68.1)	0.235	0.65	(0.34–1.25)		
Body temperature (median〔IQR^*c*^〕）	37.8	(36.8–38.7)	38.3	(37.1–39.1)	**0.015**	0.70	(0.53–0.92)		
WBC (×10^3^/µL) (median〔IQR^*c*^〕）	6.9	(1.5–14.4)	9.7	(6.33–13.9)	**0.019**	0.97	(0.91–1.02)		
Plt (×10^4^/µL) (median〔IQR^*c*^〕）	13.9	(6–23.6)	17.3	(11.4–22.9)	0.085	0.97	(0.94–1.01)		
CRP (mg/dL) (median〔IQR^*c*^〕）	11.1	(3.3–20.2)	6.8	(2.1–16.5)	0.065	1.03	(0.99–1.06)		
Comorbidities									
Charlson index (median 〔IQR^*c*^〕)	2	(1–3)	2	(1–3)	0.708	1.01	(0.83–1.21)		
Diabetes mellitus	7	(14.6)	58	(28.4)	0.066	0.43	(0.18–1.01)		
Solid tumors	17	(35.4)	69	(33.8)	0.866	1.07	(0.56–2.07)		
Liver cirrhosis	1	(2.1)	14	(6.9)	0.316	0.29	(0.04–2.25)		
Collagen disease	1	(2.1)	4	(2.0)	1	1.06	(0.12–9.74)		
Kidney disease	6	(12.5)	14	(6.9)	0.232	1.94	(0.70–5.34)		
Pulmonary disease	1	(2.1)	6	(2.9)	1	0.70	(0.08–5.97)		
Hematological malignancy	6	(12.5)	12	(5.9)	0.122	2.29	(0.81–6.44)		
Immunosuppressive drug	8	(16.7)	28	(13.7)	0.647	1.26	(0.53–2.96)		
Neutropenia	5	(10.4)	3	(1.5)	**0.007**	**7.79**	**(1.79–33.84)**	**8.88**	**(1.38–57.2)**
Hemodialysis	1	(2.1)	8	(3.9)	1	0.52	(0.06–4.27)		
Source of infection									
Biliary tract/cholecystitis	6	(12.5)	82	(40.2)	**<0.001**	0.21	(0.08–0.52)		
Liver abscess	1	(2.1)	13	(6.4)	0.481	0.31	(0.04–2.45)		
Intra-abdominal	9	(18.8)	11	(5.4)	**0.005**	**4.05**	**(1.57–10.43)**	**6.25**	**(2.02–19.3)**
Intravenous catheter related	0	(0)	11	(5.4)	0.131	0			
Respiratory tract	17	(35.4)	8	(3.9)	**<0.001**	**13.44**	**(5.34–33.77)**	**10.37**	**(3.53–30.43)**
Skin and soft tissue	1	(2.1)	2	(1.0)	0.471	2.15	(0.19–24.20)		
Urinary tract	4	(8.3)	48	(23.5)	**0.018**	0.30	(0.10–0.86)		
Others	1	(2.1)	3	(1.5)	0.573	1.43	(0.15–14.01)		
Unknown	9	(18.8)	26	(12.8)	0.352	1.58	(0.69–3.64)		
Appropriate antibiotic therapy within 24 h	38	(79.2)	176	(86.3)	0.261	0.60	(0.27–1.35)	0.52	(0.19–1.41)
Hospital-acquired infection	23	(47.9)	61	(29.9)	**0.026**	**2.16**	**(1.14–4.09)**	1.57	(0.70–3.52)
Continuous bacteremia	3	(6.3)	7	(3.4)	0.408	1.88	(0.47–7.54)	0.73	(0.12–4.45)
Disseminated infection	0	(0)	7	(3.4)	0.353	0		<0.01	0.00-0.00
q-PITT score (median〔IQR^*c*^〕)	1	(0–2)	0	(0–1)	**<0.001**	**2.20**	**(1.62–3.00)**	**1.92**	**(1.33–2.76)**
SOFA score (median〔IQR^*c*^〕）	5	(3–11)	3	(2–5)	**<0.001**	**1.27**	**(1.16–1.41)**		
**Bacterial characteristics**									
*K. pneumoniae*	35	(72.9)	143	(70.1)	0.860	1.15	(0.57–2.32)		
*K. variicola*	12	(25.0)	48	(23.5)	0.851	1.08	(0.52–2.25)		
*K. quasipneumoniae*	1	(2.1)	13	(6.4)	0.481	0.31	(0.04–2.45)		
Presumptive hypervirulent strains^*d*^	12	(25)	28	(13.7)	0.077	2.09	(0.97–4.50)	1.40	(0.49–4.01)
Hypermucoviscous phenotype	8	(16.7)	40	(19.6)	0.838	0.82	(0.36–1.89)		
Polymicrobial	9	(18.8)	53	(26)	0.354	0.66	(0.30–1.45)		
ESBL-producing(include AmpC)	0	(0)	10	(4.9)	0.216	0			

^*a*^ Results of Fisher's exact test.

^*b*^ Factors that were significantly associated with mortality in the univariate analysis were included in the initial multivariate analysis. To further refine the model, additional clinically and microbiologically important factors were also included in the analysis.

^*c*^ IQR, interquartile range.

^*d*^ A presumptive hypervirulent strain was defined as one harboring one or more of the *peg-344*, *iucA*, or *rmpA* genes.

^*e*^ Statistically significant differences are indicated in bold.

### Antimicrobial susceptibility test

Table 5 presents the results of the antimicrobial susceptibility testing and Fisher’s exact test. Among the *K. pneumoniae* isolates analyzed, 9/178 (5.1%) were producing ESBL, while 1/178 (0.6%) were producing AmpC. Conversely, none of these mechanisms were detected in *K. variicola* or *K. quasipneumoniae* isolates.

**TABLE 5:** Results of antimicrobial susceptibility test against *K. variicola*, *K. pneumoniae,* and *K. quasipneumoniae^a^*^,*d*^

Antimicrobial agents^*c*^	Number of susceptible strains *n,* (%)						*P*-value^*b*^
	*K. variicola*		*K. pneumoniae*		*K. quasipneumoniae*		
Piperacillin	52/60	(86.7)	97/178	(54.5)	3/14	(21.4)	**<0.001**
Ampicillin/sulbactam	60/60	(100)	150/178	(84.3)	14/14	(100)	**<0.001**
Amoxicillin/clavulanic acid	60/60	(100)	155/167	(92.8)	14/14	(100)	0.064
Piperacillin/tazobactam	60/60	(100)	169/178	(94.9)	14/14	(100)	0.219
Cefazolin	59/60	(98.3)	162/178	(91.0)	14/14	(100)	0.123
Cefaclor	60/60	(100)	165/178	(92.7)	14/14	(100)	0.062
Cefmetazole	60/60	(100)	177/178	(99.4)	14/14	(100)	1
Cefotaxime	60/60	(100)	168/178	(94.4)	14/14	(100)	0.179
Ceftriaxone	60/60	(100)	168/178	(94.4)	14/14	(100)	0.179
Ceftazidime	60/60	(100)	171/178	(96.1)	14/14	(100)	0.219
Cefpodoxime proxetil	59/59	(100)	156/167	(93.4)	14/14	(100)	0.101
Cefepime	60/60	(100)	171/178	(96.1)	14/14	(100)	0.219
Imipenem	60/60	(100)	176/178	(98.9)	14/14	(100)	1
Meropenem	60/60	(100)	177/178	(99.4)	14/14	(100)	1
Aztreonam	60/60	(100)	172/178	(96.6)	14/14	(100)	0.534
Amikacin	60/60	(100)	178/178	(100)	14/14	(100)	
Gentamicin	60/60	(100)	170/178	(95.5)	14/14	(100)	0.310
Minocycline	57/60	(95.0)	155/178	(87.1)	14/14	(100)	0.128
Ciprofloxacin	57/59	(96.6)	154/167	(92.2)	14/14	(100)	0.423
Levofloxacin	57/60	(95.0)	152/178	(85.4)	14/14	(100)	0.059
Sulfamethoxazole-trimethoprim	58/60	(96.7)	151/178	(84.8)	14/14	(100)	**0.008**
β-Lactamase-production							
Extended-spectrum β-lactamase-producing strains	0/60		9/178	(5.1)	0/14		0.219
AmpC β-lactamase-producing strains	0/60		1/178	(0.6)	0/14		1

^*a*^ According to CLSI document：M100-S33.

^*b*^ Results of Fisher's exact test.

^*c*^ Susceptible-dose-dependent was categorized as susceptible.

^*d*^ Statistically significant differences are indicated in bold.

### Multilocus sequence typing of *K. variicola* strains

**Fig 1:**
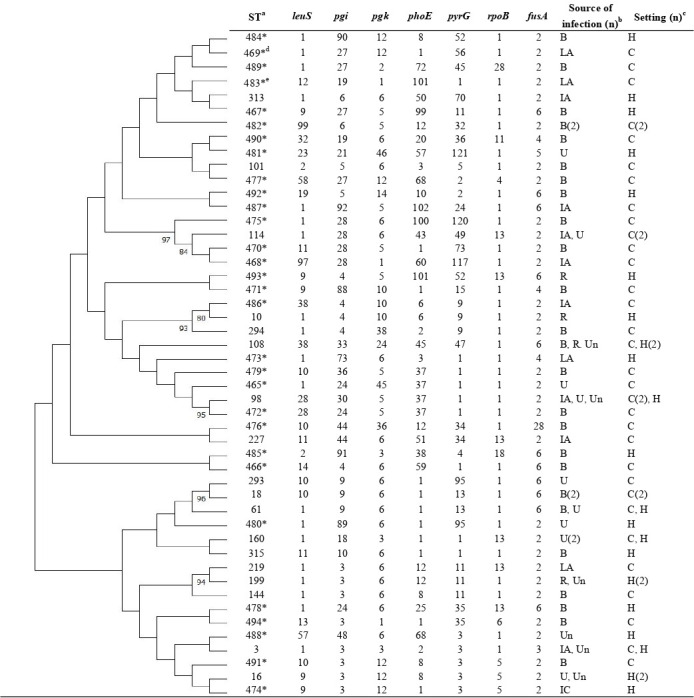
The metadata included sequence type, point mutation sites, source of infection and differences between hospital-acquired infections and community-acquired infections. ^a^Sequence types were determined according to the *K. variicola* multilocus sequence typing scheme (https://mlstkv.insp.mx/); *newly registered sequence type. ^b^B, Biliary tract; LA, liver abscess; IA, intra-abdominal; U, urinary tract; R, respiratory tract; IC, intravenous catheter related; Un, unknown. ^c^C, Community acquired; H, hospital acquired. ^d^ Presumptive hypervirulent strain (harboring *rmpA* gene). ^e^Presumptive hypervirulent strain (harboring *iucA*, *peg-344*, *rmpA* genes).

Figure 1 presents the MLST phylogenetic tree for 60 *K. variicola* strains. The metadata included ST, source of infection, distinction between hospital-acquired and community-acquired infections. The *K. variicola* isolates represented 48 diverse STs, of which 30 were identified as new STs. The founder ST10 and single-locus variants ST294 and ST486 were classified as clonal complex 1 (CC1).

### Genomic characteristics of p-hv *K. variicola* strains

A whole-genome sequencing analysis was performed on two genetically defined p-hv *K. variicola* strains found in this study (Table S3). These strains caused community-acquired liver abscesses, which are typical presentations of the p-hv *K. pneumoniae* species complex (Table S2).

Both the strains carried *bla*_LEN_, confirming their identity as *K. variicola*. Despite differing in STs and O-antigen genotypes, they shared a common capsular genotype (KL101). The virulence gene profile differed between the two strains, with TUM24737 exhibiting a higher virulence score (calculated based on the presence of yersiniabactin (*ybt*), colibactin (*clb*), and aerobactin (*iuc*) as described by Lam et al. [22]), compared with TUM24736.

## DISCUSSION

This study aimed to analyze 252 patients with BSI caused by *K. pneumoniae* species complex (including 60 patients with *K. variicola*) and to clarify the clinical and microbiological characteristics of *K. variicola* BSI. To the best of our knowledge, this is the largest study of *K. variicola* BSI conducted in Japan. No significant differences were observed in patient characteristics or clinical presentations between *K. variicola* and other *Klebsiella* spp. The prevalence of p-hv strains differed across *K. pneumoniae* species complex significantly. Although the prevalence of p-hv *K. variicola* was low, the identification of liver abscess suggests a potential risk associated with this species.

Discrepancies were observed between MALDI-TOF MS and multiplex PCR in identifying *Klebsiella* spp. The differences in microbiological characteristics among *K. pneumoniae* species complex observed in this study, as well as the significant differences in 30-day mortality reported in previous studies (4), highlight the necessity for accurate species identification in scientific investigations. However, we acknowledge the limitations of multiplex PCR targeting chromosomal β-lactamase genes, as illustrated by a strain identified as *K. pneumoniae* by MALDI-TOF MS but negative by PCR, potentially due to gene loss. Furthermore, distinguishing *K. variicola* from *K. quasipneumoniae* can be challenging when *K. variicola* harbors *bla*_OKP_ instead of *bla*_LEN_ (23). Nevertheless, we recognize the practical challenges faced in gene-based identification during routine identification performed by clinical laboratories. Therefore, despite the inability of the current library-based MALDI-TOF MS to accurately identify *K. quasipneumoniae*, we maintain that MALDI-TOF MS is the most practical and effective method for routine species differentiation within the *K. pneumoniae* species complex.

The adonitol hydrolysis test detected 5 (8.3%) *K. variicola* strains. Although a previous study indicated that a negative adonitol hydrolysis test could aid in differentiating *K. variicola* from *K. pneumoniae* (24), our findings were inconsistent, suggesting that this test alone may not be definitive for species identification.

In a previous study conducted in Sweden, *K. variicola* BSI was associated with an increased mortality risk compared with BSI caused by other *Klebsiella* spp (4). Our study found no significant difference in 30-day mortality. However, patients with *K. variicola* BSI had significantly lower qPITT scores, particularly for hypothermia (temperature <36°C), cardiac arrest, and altered mental status, compared to those with other *Klebsiella* spp. BSI.

In our study, only 2 out of the 10 hypermucoviscosity phenotype *K. variicola* strains possessed the *rmpA* gene. Therefore, we conducted the string test (with a positive cutoff of >10 mm) and the mucoviscosity assay test on these strains ( Table S1). The string test (>10 mm) was positive in 7/10 strains, and the mucoviscosity value was >0.15 in all these strains. Given that classical *K. pneumoniae* strains typically exhibit mucoviscosity values below 0.15 (25), we conclude that the hypermucoviscosity phenotype *K. variicola* isolates in this study demonstrate hypermucoviscous properties through a mechanism independent of the *rmpA* gene. Reports of *rmpA*-negative hypermucoviscosity in *K. variicola* are scarce, making the prevalence observed in this study a notable finding. The discovery of *rmpA*-deficient hypermucoviscous *K. variicola* provides critical insights into the phenotypic diversity and genetic plasticity of this species. The emergence of hypervirulent *K. variicola* strains along with the global spread of multidrug-resistant Enterobacterales poses a significant public health threat (11, 26). Although no multidrug-resistant *K. variicola* strains were detected in this study, p-hv strains were detected in 3.3% of the isolates. These strains, isolated from patients with diabetes exhibiting community-acquired liver abscesses, highlight the potential severity of *K. variicola* infections. Although previous studies have reported liver abscesses caused by *K. variicola*, no specific ST or capsular genotype has been linked to an epidemic, unlike the hypervirulent *K. pneumoniae* K1-ST23 (22, 27–30). Notably, the p-hv *K. variicola* strains in this study shared the KL101 capsular genotype despite variations in STs and O-antigen genotypes. In one patient (TUM24737), the infection disseminated, causing brain and iliopsoas abscesses. To the best of our knowledge, this is the first report in Japan of a *K. variicola* isolate harboring the same virulence genes as hypervirulent *K. pneumoniae* and causing disseminated liver abscesses, and the first to genetically characterize such an isolate using whole-genome sequencing. This case highlights the potential severity of p-hv *K. variicola* infections and emphasizes the need for further research on their molecular characteristics to better understand their relationship with the clinical features and pathogenicity.

The findings of this study suggest that the hypermucoviscous phenotype, as determined by the string test, may not be directly correlated with the clinical presentation. The accuracy of the string test in predicting hypervirulent *K. pneumoniae* has been investigated in previous studies (5), and the present study supports this notion. The positive predictive value of the string test for identifying p-hv strains was only 31.3%, indicating a high risk of false positives (data not shown). Therefore, microbiology laboratories and physicians should cautiously interpret positive string test results. In this study, the test was conducted using strains maintained in skim milk medium. Therefore, the potential influence of the test conditions on the results cannot be excluded.

Although the *K. pneumoniae* MLST scheme is commonly used in the epidemiological analyses of *K. pneumoniae* species complex, issues have been raised regarding *K. variicola* (3, 23). In this study, we adopted the *K. variicola* MLST scheme (21). The *K. variicola* isolates were non-clonal and had 49 diverse STs (30 of which were newly registered); no specific trend was observed in the source or setting of infection. Although CC1 has the highest distribution, only two strains (ST10 and ST486) were detected in this study (3).

This study has some limitations, including geographic restrictions to a specific region in Japan and the small sample size of p-hv *K. variicola* cases. These limitations underscore the need for larger studies to comprehensively evaluate the clinical and microbiological characteristics of *K. variicola* BSI.

In conclusion, no significant clinical differences were found between *K. variicola* and the other *Klebsiella* spp. BSI as opposed to previous studies. Although only a minority (3.3%) of *K. variicola* isolates harbored cardinal virulence genes, suggesting a potentially lower virulence compared with *K. pneumoniae*, the identification of two cases of community-acquired liver abscess caused by hypervirulent *K. variicola* highlights the potential clinical significance of this species. Given the lack of definitive data regarding the prevalence of antimicrobial-resistant and hypervirulent *K. variicola*, continued surveillance of this pathogen is crucial.

## Data Availability

The genomic sequences of the *K. variicola* strains were deposited in the National Center for Biotechnology Information database (accession numbers: JBFQJF000000000 [TUM24736] and JBFQJE000000000 [TUM24737]).

## SUPPLEMENTAL MATERIAL

Supplemental material spectrum.03017-24-s0001.docx Tables S1 to S3.

ASM does not own the copyrights to Supplemental Material that may be linked to, or accessed through, an article. The authors have granted ASM a non-exclusive, world-wide license to publish the Supplemental Material files. Please contact the corresponding author directly for reuse.
