# Supplementary material for: Molecular epidemiology and clinical features of Klebsiella variicola bloodstream infection compared with infection with other Klebsiella pneumoniae species complex strains
Source: Microbiol Spectr. 2025 Apr 25;13(6):e03017-24. doi: 10.1128/spectrum.03017-24 (PMC12131774; doi:10.1128/spectrum.03017-24)
Supplement: Supplemental material — Tables S1 to S3. [file spectrum.03017-24-s0001.docx]

**Table S1.** Primers used in this study

| **Gene^a^** | **Primer sequence (5′–3′)** | **Size product**  **(bp)** | **Annealing temperature** | **Extension time** | **Reference** |
| --- | --- | --- | --- | --- | --- |
| *bla*_SHV_ | F: GCTGGCGGTACACGCCAGCCCG | 995 | 55 | 10 | 14 |
| *bla*_LEN_ | F: CACGCTGCGYAAACTACTGACYGCGCAGCA | 485 |  |  |  |
| *bla*_OKP_ | F: GGCCGGYGAGCGGGGCTCA | 348 |  |  |  |
| *deo*R | R: AGAAGCATCCTGCTGTGCG |  |  |  |  |
| *peg-344* | F: CTTGAAACTATCCCTCCAGTC | 508 | 53 | 40 | 19 |
|  | R: CCAGCGAAAGAATAACCCC |  |  |  |  |
| *iucA* | F: AATCAATGGCTATTCCCGCTG | 239 | 59 | 30 |  |
|  | R: CGCTTCACTTCTTTCACTGACAGG |  |  |  |  |
| *rmpA* | F: GAGTAGTTAATAAATCAATAGCAAT | 332 | 50 | 40 |  |
|  | R: CAGTAGGCATTGCAGC |  |  |  |  |

^a^The forward primers *bla*_SHV_, *bla*_LEN_, and *bla*_OKP_ were used in combination with *deo*R reverse primer in the multiplex polymerase chain reaction

**Table S2.** Characteristic of patients with hypermucoviscous phenotype *K. variicola* bloodstream infection

| **Characteristic** | **1** | **2** | **3** | **4** | **5** | **6** | **7** | **8** | **9** | **10** |
| --- | --- | --- | --- | --- | --- | --- | --- | --- | --- | --- |
| Sequence type | 484 | 486 | 160 | 488 | 18 | 465 | 469 | 482 | 482 | 483 |
| Age | 71 | 95 | 91 | 68 | 82 | 92 | 60 | 71 | 79 | 58 |
| Sex^a^ | M | M | F | M | M | F | M | M | M | M |
| Setting^b^ | C | C | C | H | C | C | C | C | C | C |
| Mucoviscosity assay | 0.31 | 0.21 | 0.19 | 0.15 | 0.25 | 0.23 | 0.23 | 0.20 | 0.16 | 0.33 |
| String test (5 mm / 10 mm) | + / + | + / - | + / - | + / - | + / + | + / + | + / + | + / + | + / + | + / + |
| Virulence gene | - | - | - | - | - | - | *rmpA* | - | - | *iucA, peg-344, rmpA* |
| Charlson score | 0 | 0 | 1 | 5 | 1 | 0 | 1 | 5 | 0 | 1 |
| Diabetes mellitus | - | - | - | - | + | - | + | - | - | + |
| Source^c^ | B | IA | U | Un | B | U | LA | B | B | LA |
| SOFA score | 4 | 6 | 5 | 6 | 3 | 2 | 5 | 2 | 6 | 6 |
| q-PITT score | 1 | 1 | 1 | 1 | 0 | 0 | 0 | 0 | 1 | 1 |
| 30-day mortality | - | + | - | - | - | - | - | - | - | - |
| Polymicrobial infection | - | - | - | - | - | - | - | - | - | - |
| Endogenous endophthalmitis | - | - | - | - | - | - | - | - | - | - |
| Disseminated infection | - | - | - | - | - | - | - | - | - | + |
| Continuous bacteremia | - | - | - | - | - | - | - | - | - | - |

^a^ M, male; F, Female

^b^ C, community-acquired infection; H, hospital-acquired infection

^c^ B, Biliary tract; LA, Liver abscess; IA, Intra-abdominal; U, Ulinary tract; Un, Unknown.

**Table S3.** Genomic characteristics of hypervirulent *K. variicola* strains

| **Strain** | **TUM24736** | **TUM24737** |
| --- | --- | --- |
| Capsular genotype | KL101 | KL101 |
| O-antigen genotype | O3/O3a | O5 |
| Sequence type | 469 | 483 |
| Antimicrobial resistance genes | *bla*_LEN-16_ | *bla*_LEN-24_ |
| Virulence genes | *iroBCDN, irp1, irp2, kfuC, mrkABDFHI, rmpA,* and *ybtAEPQSTUX* | *iroBCDN, iucABCD, iutA, kfuAC, mrkABDFH, rmpA,* and *rmpA2* (frameshift) |
| Virulence score^a^ | 1  Yersiniabactin only | 3  Aerobactin and/or salmochelin only (without yersiniabactin or colibactin) |
| Accession number | JBFQJF000000000 | JBFQJE000000000 |

^a^ Virulence scores were determined by the presence of yersiniabactin (*ybt*), colibactin (*clb*), and aerobactin (*iuc*), as described in Lam et al. (22).
